# Water as a Medicine

**Published:** 1891-09

**Authors:** 


### WATER AS A MEDICINE.

The human body is constantly undergoing tissue change. Worn out particles are cast aside and eliminated from the system, while the new are ever being formed, from the inception of life to its close.

Water has the power of increasing these tissue changes, which multiplies the waste products, but at the same time they are renewed by its agency, giving rise to increased appetite, which in turn provides fresh nutriment. Persons but little accustomed to drink water are liable to have the waste products formed faster than they are removed. Any obstruction to the free working of natural laws at once produces disease, which if once firmly seated, requires both time and money to cure.

People accustomed to rise in the morning weak and languid will find the cause in the imperfect secretion of wastes, which many times may be remedied by drinking a full tumbler of water before retiring. This very materially assists in the process during the night, and leaves the tissues fresh and strong, ready for the active work of the day.

Hot water is one of our best remedial agents.

A hot bath on going to bed, even in the hot nights of summer, is a better reliever of insomnia than many drugs.

Inflamed parts will subside under the continual poulticing of real hot water.

Very hot water, as we all know, is a prompt checker of bleeding, and besides, if it is clean, as it should be, it aids in sterilizing our wound.

A riotous or rotten stomach will nearly always gratefully receive a glass or more of hot water.

